# Complete mitochondrial genomes of the ‘intermediate form’ of *Fasciola* and *Fasciola gigantica*, and their comparison with *F. hepatica*

**DOI:** 10.1186/1756-3305-7-150

**Published:** 2014-03-31

**Authors:** Guo-Hua Liu, Robin B Gasser, Neil D Young, Hui-Qun Song, Lin Ai, Xing-Quan Zhu

**Affiliations:** 1 State Key Laboratory of Veterinary Etiological Biology, Key Laboratory of Veterinary Parasitology of Gansu Province, Lanzhou Veterinary Research Institute, Chinese Academy of Agricultural Sciences, Lanzhou, Gansu Province, PR China; 2 Faculty of Veterinary Science, The University of Melbourne, Parkville, Victoria, Australia; 3 College of Veterinary Medicine, Hunan Agricultural University, Changsha, Hunan Province, PR China; 4 National Institute of Parasitic Diseases, Chinese Center for Disease Control and Prevention, Shanghai, PR China

**Keywords:** Liver fluke, *Fasciola* spp, Mitochondrial genome, Phylogenetic analysis

## Abstract

**Background:**

Fascioliasis is an important and neglected disease of humans and other mammals, caused by trematodes of the genus *Fasciola. Fasciola hepatica* and *F. gigantica* are valid species that infect humans and animals, but the specific status of *Fasciola* sp. (‘intermediate form’) is unclear.

**Methods:**

Single specimens inferred to represent *Fasciola* sp. (‘intermediate form’; Heilongjiang) and *F. gigantica* (Guangxi) from China were genetically identified and characterized using PCR-based sequencing of the first and second internal transcribed spacer regions of nuclear ribosomal DNA. The complete mitochondrial (mt) genomes of these representative specimens were then sequenced. The relationships of these specimens with selected members of the Trematoda were assessed by phylogenetic analysis of concatenated amino acid sequence datasets by Bayesian inference (BI).

**Results:**

The complete mt genomes of representatives of *Fasciola* sp. and *F. gigantica* were 14,453 bp and 14,478 bp in size, respectively. Both mt genomes contain 12 protein-coding genes, 22 transfer RNA genes and two ribosomal RNA genes, but lack an *atp*8 gene. All protein-coding genes are transcribed in the same direction, and the gene order in both mt genomes is the same as that published for *F. hepatica*. Phylogenetic analysis of the concatenated amino acid sequence data for all 12 protein-coding genes showed that the specimen of *Fasciola* sp. was more closely related to *F. gigantica* than to *F. hepatica*.

**Conclusions:**

The mt genomes characterized here provide a rich source of markers, which can be used in combination with nuclear markers and imaging techniques, for future comparative studies of the biology of *Fasciola* sp. from China and other countries.

## Background

Food-borne trematodiases are an important group of neglected parasitic diseases. More than 750 million people are at risk of such trematodiases globally [1,2]. Fascioliasis is caused by liver flukes of the genus *Fasciola*, and has a significant adverse impact on both human and animal health worldwide [3]. Human fascioliasis is caused by the ingestion of freshwater plants or water contaminated with metacercariae of *Fasciola*[4]. It is estimated that millions of people are infected worldwide, and more than 180 million people are at risk of this disease worldwide [5]. To date, no vaccine is available to prevent fascioliasis. Fortunately, this disease can be treated effectively using triclabendazole [6], but there are indications of resistance developing against this compound [7].

The Fasciolidae is a family of flatworms and includes the genus *Fasciola*. Both *F. hepatica* and *F. gigantica*, which commonly infect livestock animals and humans (as definitive hosts), are recognized as valid species [8]. The accurate identification of species and genetic variants is relevant in relation to studying their biology, epidemiology and ecology, and also has applied implications for the diagnosis of infections. Usually, morphological features, such as body shape and perimeter as well as length/width ratio, are used to identify adult worms of *Fasciola*[9]. However, such phenotypic criteria are unreliable for specific identification and differentiation, because of considerable variation and/or overlap in measurements between *F. hepatica* and *F. gigantica*[10].

Due to these constraints, various molecular methods have been used for the specific identification of *Fasciola* species and their differentiation [5]. For instance, PCR-based techniques using genetic markers in nuclear ribosomal (r) and mitochondrial (mt) DNAs have been widely used [11-13]. The sequences of the first and second internal transcribed spacers (ITS-1 and ITS-2 = ITS) of nuclear rDNA have been particularly useful for the specific identification of *F. hepatica* and *F. gigantica*, based on a consistent level of sequence difference (1.2% in ITS-1 and 1.7% in ITS-2) between them and much less variation within each species [11,14]. Nonetheless, studies in various countries, including China [5], Iran [15], Japan [16], Korea [14], Spain [17] and Tunisia [18], have shown that some adult specimens of *Fasciola* sp., which are morphologically similar to *F. gigantica*[10], are characterized by multiple sequence types (or “alleles”) of ITS-1 and/or ITS-2, reflected in a mix between those of *F. hepatica* and *F. gigantica*[11,12]. Some authors [19-21] have suggested that such specimens (sometimes called ‘intermediate forms’) represent hybrids of *F. hepatica* and *F. gigantica*.

In the present study, we undertook an independent, genetic comparison of *Fasciola* sp. (i.e. ‘intermediate form’) and *F. gigantica* with *F. hepatica*. To do this, we characterized the mt genomes of individual specimens of *Fasciola* sp. and *F. gigantica* whose identity was defined based on their ITS-1 and/or ITS-2 sequences, and assessed their relationships by comparison with *F. hepatica* and various other trematodes using complete, inferred mt amino acid sequence data sets.

## Methods

### Ethics statement

This study was approved by the Animal Ethics Committee of Lanzhou Veterinary Research Institute, Chinese Academy of Agricultural Sciences (Permit code. LVRIAEC2012-006). Adult specimens of *Fasciola* were collected from bovids, in accordance with the Animal Ethics Procedures and Guidelines of the People's Republic of China.

### Parasites and isolation of total genomic DNA

Adult specimens of *Fasciola* sp. were collected from the liver of a dairy cow (*Bos taurus*) in Heilongjiang province, China. Adult specimens of *F. gigantica* were collected from the liver of a buffalo (*Bubalus bubalis*) in Guangxi province, China. The worms were washed extensively in physiological saline, fixed in ethanol and then stored at −20°C until use. Single specimens were identified as *Fasciola* sp. or *F. gigantica* based on PCR-based sequencing of the ITS-1 and ITS-2 rDNA regions [11,12].

### Long-range PCR-based sequencing of mt DNA

To obtain some mt gene sequence data for primer design, regions (400–500 bp) of the *cox*1 and *nad*4 genes were PCR-amplified and sequenced using relatively conserved primers JB3/JB4.5 and ALF/ALR [13,22], respectively. Using BigDye terminator v.3.1 chemistry (Applied Biosystems, Weiterstadt, Germany), the amplicons were sequenced in both directions in a PRISM 3730 sequencer (ABI, USA). After sequencing regions of the *cox*1 and *nad*4 genes of both *Fasciola* sp. and *F. gigantica*, two internal pairs of conserved primers were designed (Table 1). These pairs were then used to long PCR-amplify the complete mt genome [23] in two overlapping fragments (*cox*1-*nad*4; ~9 kb and, *nad*4-*cox*1 = ~6 kb) from a proportion of total genomic DNA (10–20 ng) from one individual of *Fasciola* sp. and another of *F. gigantica*. The cycling conditions used were 92°C for 2 min (initial denaturation), then 92°C for 10 s (denaturation), 58–63°C for 30 s (annealing), and 60°C for 5 min (extension) for 5 cycles, followed by 92°C for 2 min, 92°C for 10 s, 58–63°C for 30 s, and 66°C for 5 min for 20 cycles, and a final extension at 66°C for 10 min. Each amplicon, which represented a single band in a 0.8% (w/v) agarose gel, following electrophoresis and ethidium-bromide staining [23], was column-purified and then sequenced using a primer-walking strategy [24].

**Table 1:** **Sequences of primers used to amplify mt DNA regions from *Fasciola *** **spp.**

**Primer**	**Sequence (5’ to 3’)**
*F. gigantica*	
FGCF1	TGTTTACTATTGGTGGGGTTACTGGT
FGNR1	CAAACCCTACAGAACTATCCCTCCAA
FGNF1	GTTATGGGATTCAGTCTTGGAGGGAT
FGCR1	CGTATCCAAAAGAGAAGCAGAAAGCA
*Fasciola* sp.	
FZCF1	GGGTTACTGGTATTATGCTTTCTGCT
FZNR1	CCCTACAGAACTATCCCTCCAAGACT
FZNF1	GGTGGTATTATGGGCAGTTATGGGAT
FZCR1	CAGAAAGCATAATACCAGTAACCCCA

### Sequence analyses

Sequences were manually assembled and aligned against each other, and then against the complete mt genome sequences of 11 other trematodes (see section on Phylogenetic analysis) using the program Clustal X 1.83 [25] and manual adjustment, in order to infer gene boundaries. Open-reading frames (ORFs) were established using the program ORF Finder (http://www.ncbi.nlm.nih.gov/gorf/gorf.html), employing the trematode mt code, and subsequently compared with those of *F. hepatica*[26]. Translation initiation and termination codons were identified based on comparisons with those of *F. hepatica*[26]. The secondary structures of 22 tRNA genes were predicted using tRNAscan-SE [27] with manual adjustment [28], and rRNA genes were predicted by comparison with those of *F. hepatica*[26].

### Sliding window analysis of nucleotide variation

To detect variable nucleotide sites, pairwise alignments of the complete genomes, including tRNAs and all intergenic spacers, were performed using Clustal X 1.83. The complete mt genome sequences of *Fasciola* sp. and *F. gigantica* were aligned with that published previously for *F. hepatica* (NC_002546) [26], and sliding window analysis was conducted using DnaSP v.5 [29]. A sliding window of 300 bp (in 10 bp overlapping steps) was used to estimate nucleotide diversity Pi (π) across the alignment. Nucleotide diversity was plotted against mid-point positions of each window, and gene boundaries were identified.

### Phylogenetic analysis

The amino acid sequences conceptually translated from individual genes of the mt genomes of each *Fasciola* sp. and *F. gigantica* were concatenated. For comparative purposes, amino acid sequences predicted from published mt genomes of selected members of the subclass Digenea, including *F. hepatica* (NC_002546) [26] [Fasciolidae]; *Clonorchis sinensis* (GeneBank accession no. FJ381664), *Opisthorchis felineus* (EU921260) [30] and *O. viverrini* (JF739555) [31] [family Opisthorchiidae]; *Paragonimus westermani* (NC_002354) [Paragonimidae]; *Trichobilharzia regenti* (NC_009680) [32], *Orientobilharzia turkestanicum* (HQ283100) [33], *Schistosoma mansoni* (NC_002545) [34], *S. japonicum* (HM120846) [35], *S. mekongi* (NC_002529) [34], *S. spindale* (DQ157223) [36] and *S. haematobium* (DQ157222) [35] [Schistosomatidae], were also included in the analysis. A sequence representing *Gyrodactylus derjavinoides* (accession no. NC_010976) was included as an outgroup [37]. All amino acid sequences were aligned using the program MUSCLE [38] and subjected to phylogenetic analysis using Bayesian inference (BI), as described previously [39,40]. Phylograms were displayed using the program Tree View v.1.65 [41]. In addition, all publicly available sequences of NADH dehydrogenase subunit 1 gene (*nad*1) of *Fasciola* sp.. *F. gigantica* and *F. hepatica* were aligned (over a consensus length of 359 bp) using MUSCLE, the alignment was modified manually, and then subjected to phylogenetic analysis by BI, applying the General Time Reversible (GTR) model. Nodal support values for the final phylogram were determined from the final 75% of trees obtained using a sample frequency of 100. The analysis was performed until the potential scale reduction factor approached 1 and the average standard deviation of split frequencies was less than 0.01. An *nad*1 sequence of *Fascioloides magna* was used as an outgroup in phylogenetic analysis.

## Results

### Identity of the two liver flukes, and features of the mt genomes

The ITS-1 and ITS-2 sequences (GenBank accession no. KF543341) of the specimen of *Fasciola* sp. from Heilongjiang province were the same as that of an ‘intermediate form’ of *Fasciola* from China (AJ628428, AJ557570 and AJ557571) reported previously [11,12], which is characterized by polymorphic positions at 10 positions in ITS-1 and ITS-2 (Additional file 1: Figure S1; Table 2). Based on these key polymorphic positions (cf. [11,12]), this specimen of *Fasciola* sp. from China was inferred to be a hybrid between *F. gigantica* and *F. hepatica.* The ITS-1 and ITS-2 sequences of the *F. gigantica* sample (accession no. KF543340) from Guangxi province were consistent with that of the same species from Niger (AM900371) and did not have any polymorphic positions (Table 2).

**Table 2:** **Comparison of nucleotides at variable positions in ITS-1 and ITS-2 rDNA sequences of *Fasciola *** **from different geographical locations**

**Species**	**Locations**	Variable positions in ITS-1 and ITS-2 sequences^*^										**Accession nos.**
		**18**	**108**	**202**	**280**	**300**	**791**	**815**	**854**	**860**	**911**	
*F. hepatica*	China	C	A	C	T	C	T	T	C	C	T	JF708026
	France	C	A	C	T	C	T	T	C	C	T	JF708034
	Iran	C	A	C	T	C	T	T	C	C	T	JF432072
	Niger	C	A	C	T	C	T	T	C	C	T	AM850107
	Spain	C	A	C	T	C	T	T	C	C	T	JF708036
*F. gigantica*	Burkina Faso	T	T	T	A	T	C	C	T	T	-	AJ853848
	China	T	T	T	A	T	C	C	T	T	-	JF496709
	Niger	T	T	T	A	T	C	C	T	T	-	AM900371
	Present study	T	T	T	A	T	C	C	T	T	-	KF543340
*Fasciola* sp.	China	C/T	A/T	C/T	T/A	C/T	T/C	T/C	C/T	C/T	T/-	AJ628428, AJ557570, AJ557571
	China, Japan	C	A	C	T	C	T	T	C	C	T	AB385611, AB010978
	Present study	C/T	A/T	C/T	T/A	C/T	T/C	T/C	C/T	C/T	T	KF543341

^*^Sequence positions were determined by comparison with that of a previous study [11]. Sequences include ITS-1 (polymorphic positions 18, 108, 202, 280, 300), 5.8S rDNA and ITS-2 (polymorphic positions 791, 815, 854, 860, 911).

**Figure 1:**
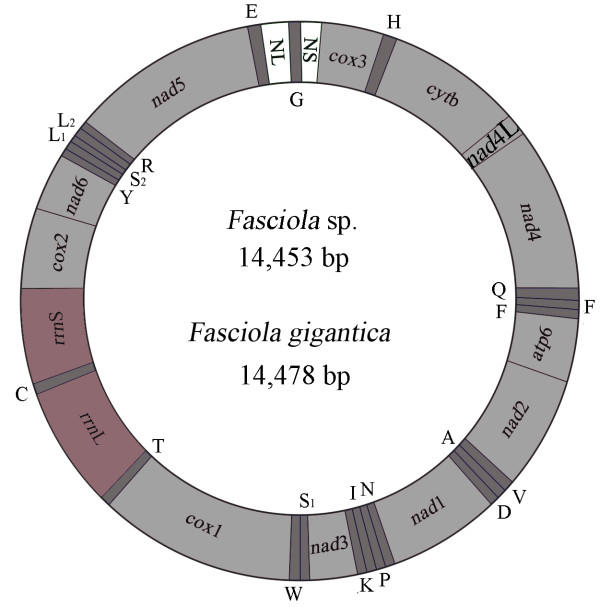
**Structure of the mitochondrial genomes of *Fasciola *sp. and *Fasciola gigantica.*** Genes are designated according to standard nomenclature [26], except for the 22 tRNA genes, which are designated using one-letter amino acid codes, with numerals differentiating each of the two leucine- and serine-specifying tRNAs (L1 and L2 for codon families CUN and UUR, respectively; S1 and S2 for codon families AGN and UCN, respectively). Large non-coding region (NS); small non-coding region (NL).

The complete mt genome sequences representing *Fasciola* sp. (GenBank accession no. KF543343) and *F. gigantica* (accession no. KF543342) were 14,453 bp and 14,478 bp in size, respectively. Each mt genome contains 12 protein-coding genes (*cox*1-3, *nad*1-6, *nad*4L, *cyt*b and *atp*6), 22 transfer RNA genes and two ribosomal RNA genes (*rrn*S and *rrn*L), but lack an *atp*8 gene (Figure 1). The mt genome arrangement of the two flukes is the same as that of *F. hepatica*[26], but as expected, distinct from *Schistosoma* spp. [36]. All genes are transcribed in the same direction and have a high A + T content (62.7%). The AT-rich regions of both mt genomes are located between tRNA-Glu and tRNA-Gly, and tRNA-Gly and *cox*3.

### Annotation

For the two liver flukes, the protein-coding genes were in the following order: *nad*5 > *cox*1 > *nad*4 > *cyt*b > *nad*1 > *nad*2 > *cox*3 > *cox*2 > *atp*6 > *nad*6 > *nad*3 > *nad*4L, and the lengths of the all protein-coding genes are the same for *Fasciola* sp. and *F. gigantica* (Table 3). The inferred nucleotide and amino acid sequences of each of the 12 mt proteins of two liver flukes were compared. A total of 3,356 amino acids are encoded in the both mt genomes. All protein-coding genes have ATG, TTG or GTG as their initiation codon (Table 3). All protein-coding genes have TAG as their termination codon, except for *cox*3 and *nad*3, which have TAA in *Fasciola* sp. (Table 3). No abbreviated stop codons, such as TA or T, were detected. Twenty-two tRNA genes were predicted from the mt genomes of the two liver flukes, and varied from 55 to 69 bp in size. Of all tRNA genes, 20 can be folded into the conventional four-arm cloverleaf structures. The tRNA-tRNA-Ser^(UCN)^ and tRNA-Ser^(AGN)^ show unorthodox structures; their D-arms are unpaired and replaced by the loops of 8–11 bp.

**Table 3:** **The organization of the mt genomes of *Fasciola *** **sp., *Fasciola gigantica *** **and *F. hepatica***

**Genes**	**Positions and nt sequence lengths (bp)**			**Ini/Ter codons**		
	***Fasciola *** **sp.**	***Fasciola gigantica***	***Fasciola hepatica***	***Fasciola *** **sp.**	***Fasciola gigantica***	***Fasciola hepatica***
*cox*3	1-642	1-642	1-642	ATG/TAA	ATG/TAG	ATG/TAG
tRNA-His	650 -713 (64)	650-713 (64)	650-713 (64)			
*cyt*b	715-1827	715-1827 (62)	715-1827	ATG/TAG	ATG/TAG	ATG/TAG
*nad*4L	1836-2108	1836-2108	1836-2108	GTG/TAG	GTG/TAG	GTG/TAG
*nad*4	2069-3337	2069-3337	2069-3340	GTG/TAG	GTG/TAG	GTG/TAA
tRNA-Gln	3339-3404 (66)	3339-3404 (66)	3342-3404 (63)			
tRNA-Phe	3420-3484 (65)	3417-3481 (65)	3417-3482 (66)			
tRNA-Met	3491-3556 (66)	3488-3553 (66)	3494-3561 (68)			
*atp*6	3557-4075	3554-4072	3562-4080	ATG/TAG	ATG/TAG	ATG/TAG
*nad*2	4088-4954	4085-4951	4093-4959	ATG/TAG	ATG/TAG	ATG/TAG
tRNA-Val	4959-5021 (63)	4957-5020 (64)	4965-5027 (63)			
tRNA-Ala	5035-5099 (65)	5035-5099 (65)	5042-5104 (63)			
tRNA-Asp	5103-5167 (65)	5103-5167 (65)	5107-5172 (66)			
*nad*1	5171-6073	5171-6073	5176-6078	GTG/TAG	GTG/TAG	GTG/TAG
tRNA-Asn	6079-6146 (68)	6084-6153 (70)	6089-6158 (70)			
tRNA-Pro	6152-6220 (69)	6163-6230 (68)	6168-6234 (67)			
tRNA-Ile	6221-6282 (62)	6231-6292 (62)	6235-6296 (62)			
tRNA-Lys	6287-6352 (66)	6297-6363 (67)	6301-6367 (67)			
*nad*3	6353-6709	6364-6720	6368-6724	ATG/TAG	ATG/TAG	ATG/TAG
tRNA-Ser^UCN^	6714-6768 (55)	6725-6780 (56)	6731-6788 (58)			
tRNA-Trp	6771-6833 (63)	6790-6852 (63)	6796-6858 (63)			
*cox*1	6837-8378	6865-8397	6871-8403	GTG/TAG	GTG/TAG	ATG/TAG
tRNA-Thr	8391-8458 (68)	8419-8486 (68)	8420-8488 (69)			
*rrn*L	8460-9445	8488-9473	8489-9475			
tRNA-Cys	9446-9510 (65)	9474-9538 (65)	9476-9538 (63)			
*rrn*S	9511-10279	9539-10309	9539-10304			
*cox*2	10280-10882	10310-10912	10305-10907	ATG/TAA	ATG/TAG	ATG/TAG
*nad*6	10929-11381	10959-11411	10950-11402	ATG/TAG	ATG/TAG	ATG/TAG
tRNA-Tyr	11389-11445 (57)	11419-11475 (57)	11411-11467 (67)			
tRNA-Leu^CUN^	11456-11520 (65)	11486-11550 (65)	11478-11543 (66)			
tRNA-Ser^AGN^	11521-11579 (59)	11551-11607 (57)	11542-11603 (62)			
tRNA-Leu^UUR^	11588-11651 (64)	11616-11678 (63)	11609-11673 (64)			
tRNA-Arg	11653-11718 (66)	11680-11745 (66)	11673-11738 (66)			
*nad*5	11720-13282	11747-13309	11737-13305	TTG/TAG	TTG/TAG	GTG/TAG
tRNA-Glu	13305-13372 (68)	13332-13399 (68)	13327-13395 (69)			
Short non-coding region	13373-13548 (176)	13400-13573 (174)	13396-13582 (187)			
tRNA-Gly	13549-13612 (64)	13574-13637 (64)	13583-13645 (63)			
Long non-coding region	13613-14453 (841)	13638-14478 (841)	13646-14462 (817)			

The two ribosomal RNA genes (*rrn*L and *rrn*S) of *Fasciola* sp. and *F. gigantica* were inferred based on comparisons with sequences from those of *F. hepatica*. The *rrn*L of *Fasciola* sp. and *F. gigantica* is located between tRNA-Thr and tRNA-Cys, and *rrn*S is located between tRNA-Cys and *cox*2. The length of *rrn*L is 987 bp for both *Fasciola* sp. and *F. gigantica*. The size of the *rrn*S genes is 769 bp and 771 bp for *Fasciola* sp. and *F. gigantica*, respectively. The A + T contents of *rrn*L and *rrn*S are ~ 62% and ~ 61% for *Fasciola* sp. and *F. gigantica*, respectively.

Two AT-rich non-coding regions (NCR) in the mt genomes *Fasciola* sp. and *F. gigantica* were inferred. In both mt genomes, the long NCR (841 bp) is located between the tRNA-Gly and *cox*3 (Figure 1), has an A + T content of ~53% and contains eight perfect, 86 bp tandem repeats (TR1 to TR8). The short NCR is 174–176 bp in length, is located between tRNA-Glu and tRNA-Gly (Figure 1) and has an A + T content of ~ 72%.

### Comparative mt genomic analyses of *Fasciola* sp. and *F. gigantica* with *F. hepatica*

The complete mt genome sequences representing *Fasciola* sp. and *F. gigantica* are 9 bp shorter and 16 bp longer than *F. hepatica* (14,462 bp in length) [26], respectively. A comparison of the nucleotide sequences of each mt gene, and the amino acid sequences, conceptually translated from all mt protein-encoding genes of the three flukes, is given in Table 4. Across the entire mt genome, the sequence difference was 2.6% (380 nucleotide substitutions) between *Fasciola* sp. and *F. gigantica*, 11.8% (1712 nucleotide substitutions) between *Fasciola* sp. and *F. hepatica*, and 11.8% (1714 nucleotide substitutions) between *F. gigantica* and *F. hepatica*. The difference across both nucleotide and amino acid sequences of the 12 protein-coding was 11.6% (1167 nucleotide substitutions) and 9.54% (320 amino acid substitutions) between the *Fasciola* sp. and *F. hepatica*; 11.6% (1167 nucleotide substitutions) and 9.83% (330 amino acid substitutions) between the *F. gigantica* and *F. hepatica*; and 2.8% (281 nucleotide substitutions) and 2.1% (71 amino acid substitutions) between the *Fasciola* sp. and *F. gigantica*, respectively.

**Table 4:** **Nucleotide (nt) and/or predicted amino acid (aa) sequence differences in each mt gene among *Fasciola *** **sp. (F), *Fasciola gigantica *** **(Fg) and *F. hepatica *** **(Fh) upon pairwise comparison**

**Gene/region**	**Nt sequence length**			**Nt difference (%)**			**Number of aa**			**aa difference (%)**		
	**F**	**Fg**	**Fh**	**F/Fg**	**F/Fh**	**Fg/Fh**	**F**	**Fg**	**Fh**	**F /Fg**	**F/Fh**	**Fg/Fh**
*atp*6	519	519	519	2.89	15.22	13.87	172	172	172	1.74	15.12	13.95
*nad*1	903	903	903	3.10	8.86	8.42	300	300	300	2.67	7.67	8.0
*nad*2	867	867	867	3.69	11.42	11.65	288	288	288	1.74	11.81	11.81
*nad*3	357	357	357	5.60	10.64	10.64	118	118	118	0.85	7.63	7.63
*nad*4	1269	1269	1272	3.86	13.99	13.68	422	422	423	3.08	11.58	11.11
*nad*4L	273	273	273	1.83	8.79	8.42	90	90	90	2.22	5.56	5.56
*nad*5	1563	1563	1569	1.86	13.58	14.02	520	520	522	1.35	12.45	12.45
*nad*6	453	453	453	3.97	13.91	16.34	150	150	150	7.33	8.00	14.67
*cox*1	1542	1542	1533	2.02	9.39	9.13	513	513	510	1.37	6.08	5.49
*cox*2	603	603	603	2.16	11.11	11.61	200	200	200	0.50	7.00	7.50
*cox*3	642	642	642	2.80	13.86	13.40	213	213	213	2.82	14.55	14.55
*cyt*b	1113	1113	1113	2.07	8.36	8.36	370	370	370	1.89	6.22	7.03
*rrn*S	769	771	766	1.30	11.31	11.41		-	-		-	
*rrn*L	986	986	987	1.01	9.93	10.13		-	-		-	
22 tRNAs	1413	1414	1420	2.26	10.28	10.63						

### Nucleotide variability in the mt genome among *Fasciola* sp., *F. gigantica* and *F. hepatica*

**Figure 2:**
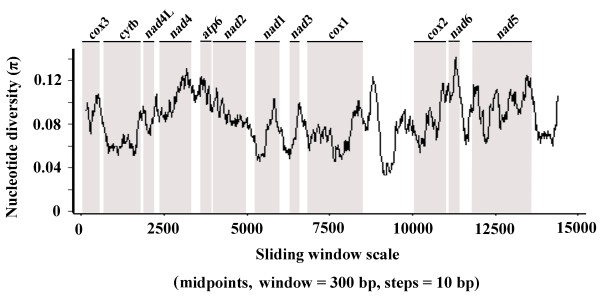
**Sliding window analysis of complete mt genome sequences of *Fasciola *sp., *Fasciola gigantica *and *F. hepatica*.** The black line indicates nucleotide diversity in a window of 300 bp (10 bp-steps). Gene regions (grey) and boundaries are indicated.

Sliding window analysis across the mt genomes of *Fasciola* sp., *F. gigantica* and *F. hepatica* provided an estimation of nucleotide diversity Pi (π) for individual mt genes (Figure 2). By computing the number of variable positions per unit length of gene, the sliding window indicated that the highest and lowest levels of sequence variability were within the genes *nad*6 and *cyt*b, respectively. Conserved regions were identified within *nad*1 and *cox*1 genes. In this study, the *cyt*b and *nad*1 genes are the most conserved protein-coding genes, and *nad*6, *nad*5 and *nad*4 are the least conserved.

### Phylogenetic analysis

**Figure 3:**
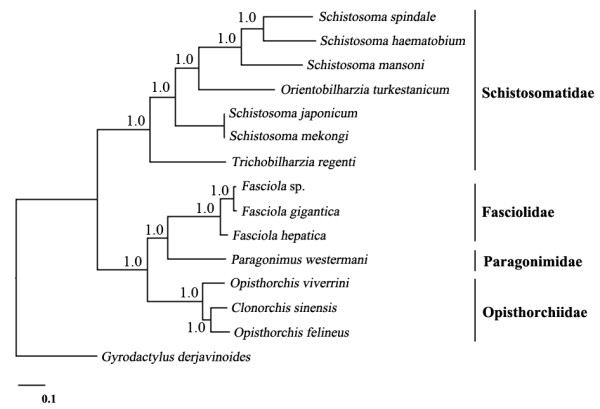
**Genetic relationships of *Fasciola *sp. with *Fasciola gigantica *and *F. hepatica*, and other trematodes.** Phylogenetic analysis of the concatenated amino acid sequence data representing 12 protein-coding genes was conducted using Bayesian inference (BI), using *Gyrodactylus derjavinoides* (NC_010976) as an outgroup.

Phylogenetic analysis of the concatenated amino acid sequence data for all 12 mt proteins (Figure 3) showed that the Fasciolidae clustered to the exclusion of representatives of the families Paragonimidae (*P. westermani*) and Opisthorchiidae (*O. viverrini*, *O. felineus* and *C. sinensis*); the Schistosomatidae clustered separately with strong nodal support (posterior probability (pp) = 1.0). Within the Fasciolidae, *Fasciola* sp. and *F. gigantica* clustered together with strong support (pp = 1.0), to the exclusion of *F. hepatica*, with the former two taxa being more closely related than either was to *F. hepatica*. In addition, phylogenetic analysis using the *nad*1 data supports clustering of the *Fasciola* sp. with aspermic *F. gigantica* x *F. hepatica* hybrids characterised previously [42] (Additional file 2: Figure S2).

## Discussion

The present comparative, genetic investigation of representative specimens of *Fasciola* sp. (i.e. the ‘intermediate form’), *F. gigantica* and *F. hepatica* using whole mt genomic and protein sequence data sets showed that *Fasciola* sp. and *F. gigantica* were more closely related than either was to *F. hepatica*. This finding was also supported by an analysis of *nad*1 sequence data (cf. Additional file 2: Figure S2). Although this evidence might suggest that *Fasciola* sp. is a population variant of *F. gigantica*, previous studies [19-21] have proposed that *Fasciola* sp. is a hybrid of *F. gigantica* and *F. hepatica.* The combined use of mtDNA (if indeed maternally inherited in fasciolids) and nuclear DNA markers [43] should assist in exploring the “hybridization/speciation” hypotheses [44]. Clearly, there is consistent evidence from various studies [11,12,14] of mixed ITS-1 and ITS-2 sequence types, representing both *F. gigantica* and *F. hepatica* among the multiple rDNA copies, within individual specimens of *Fasciola* sp. (i.e., the ‘intermediate form’). Although the number or proportion(s) of different sequence types within individual adults of *Fasciola* sp. has not yet been estimated using a mutation scanning- or cloning-based sequencing [45], the polymorphic positions in the sequences determined by direct sequencing [11,14] indicate a clear pattern of introgression between the *F. gigantica* and *F. hepatica*. Although mt genomic (11.8%) and inferred protein (9.83%) sequence differences between these two species is substantial, the explanation that *Fasciola* sp. represents a hybrid between these two recognized species seems plausible, given that the karyotypes of both diploid *F. hepatica* and *F. gigantica* are the same (2n = 20) [46,47] and that the magnitude of sequence variation (1.7%) in ITS-2 (a species marker) between *F. gigantica* and *F. hepatica* is comparable with the highest level (1.3-1.6%) in this rDNA region between some schistosome species for which hybrids (i.e. *S. haematobium × S. bovis*; *S. haematobium* × *S. guineenis*; *S. haematobium* × *S. intercalatum*) have been reported [48-50]. While hybridization seems possible, another explanation might be ITS rDNA "lineage sorting and retention of ancestral polymorphism" [51,52], but this is perhaps less likely, given a clear pattern of mixing of ITS sequences seen in *Fasciola* sp. (cf. Additional file 1: Figure S1).

In addition, polyploidy or diploidy in aspermic *Fasciola*[20] needs to be considered, and warrants future investigation. Perhaps the aspermic *Fasciola* specimens described in the literature [53] were infertile hybrids of *F. gigantica* and *F. hepatica* (in situations where both species occur in sympatry). Questions that might be addressed directly in relation to *Fasciola* sp. are: Are eggs from *Fasciola* sp. fertilized and viable? If miracidia develop and emerge from these eggs, are they infective to snails? If they do infect snails, do the ensuing adult worms (in the definitive host) contain sperm and are these worms fertile, and what is their ploidy? These questions should be addressed, and could be complemented by detailed light and transmission electron microscopic investigations of a relatively large number of adult specimens of *Fasciola* sp., *F. gigantica* and *F. hepatica* (preferably from different countries), which have been unequivocally and individually identified based on their ITS-1 and ITS-2 sequences. Such a study should pay particular attention to the morphology of the reproductive organs, sperm and oocytes, and the karyotypes of worms, and establish whether or not *Fasciola* sp. from China is polyploid and/or aspermic [20].

Moreover, although challenging, laborious and time-consuming, it would be highly informative to conduct hybridization studies *in vivo*, whereby individual miracidia from eggs from adults of each *Fasciola* sp., *F. gigantica* and *F. hepatica* would be used to infect (separately) their lymnaeid snail hosts, to raise clonal populations of cercariae and metacercariae of these three taxa, so that mixed infections (in different combinations and with mono-specific controls) could be established in, for example, sheep or goats, to attempt to cross-hybridize the three taxa in a pairwise manner. Using such an experimental design, eggs and adult worms could then be examined in detail at both the electron microscopic, karyotypic and molecular levels. Importantly, in these experiments, ITS-1 and/or ITS-2 could be used to establish the genotypes of subsamples of individuals, and mt markers derived from mt genomes determined here and of *F. hepatica* could be used to determine haplotypes and mtDNA inheritance if the cross-hybridization studies were successful. Therefore, the present markers could be employed, in combination, to establish the biological relationship of the three taxa through *in vivo* experiments, but also in the field in sympatric and allopatric populations, if they occur. Combined with the use of markers in nuclear and mt genomes, advanced genomic sequencing, optical mapping and micro-imaging techniques might assist studies of *Fasciola* sp. in China and other countries.

## Conclusion

The findings of this study provide robust genetic evidence that *Fasciola* sp. is more closely related to *F. gigantica* than to *F. hepatica*. The mtDNA datasets reported in the present study should provide useful novel markers for further studies of the taxonomy and systematics of *Fasciola* from different hosts and geographical regions.

## Competing interests

The authors declare that they have no competing interests.

## Authors’ contributions

GHL, NY, HQS and LA performed the experiments, analyzed the data and drafted parts of the manuscript. XQZ and RBG revised and edited the manuscript and funded the study. All authors read and approved the final manuscript.

## Supplementary Material

Additional file 1: Figure S1 Polymorphic positions in the internal transcribed spacer regions (ITS-1 and ITS-2) of nuclear ribosomal DNA of *Fasciola* sp. Click here for file

Additional file 2: Figure S2 Phylogenetic tree of *Fasciola* spp. inferred from the mitochondrial *nad*1 sequence data by Bayesian inference (BI). *Fascioloides magna* was used as an outgroup. Nodal support values were determined from the final 75% of trees using a sampling frequency of 100. Click here for file
