# Supplementary figures and images for: Complete mitochondrial genomes of the ‘intermediate form’ of Fasciola and Fasciola gigantica, and their comparison with F. hepatica
Source: Parasit Vectors. 2014 Mar 31;7:150. doi: 10.1186/1756-3305-7-150 (PMC3997819; doi:10.1186/1756-3305-7-150)

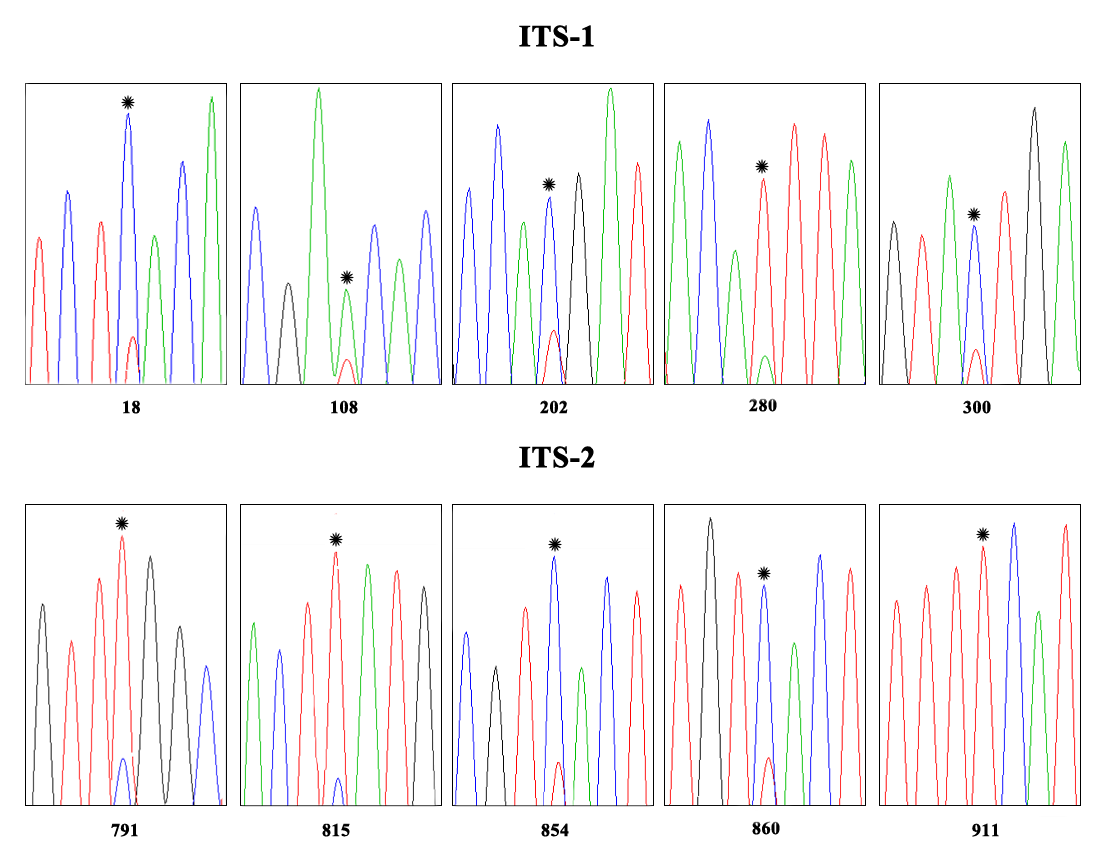

Supplement: Additional file 1: Figure S1 — Polymorphic positions in the internal transcribed spacer regions (ITS-1 and ITS-2) of nuclear ribosomal DNA of Fasciola sp. [file 1756-3305-7-150-S1.tiff]

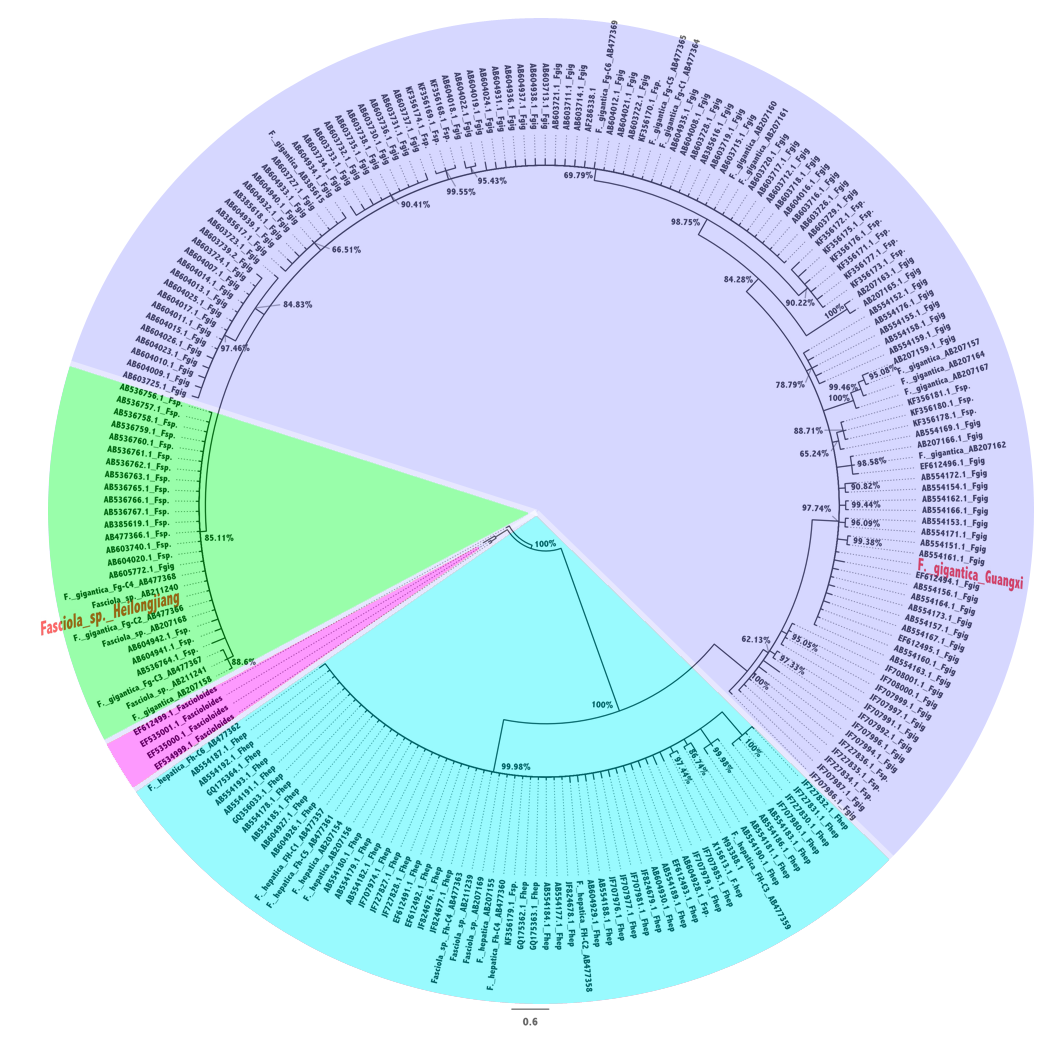

Supplement: Additional file 2: Figure S2 — Phylogenetic tree of Fasciola spp. inferred from the mitochondrial nad1 sequence data by Bayesian inference (BI). Fascioloides magna was used as an outgroup. Nodal support values were determined from the final 75% of trees using a sampling frequency of 100. [file 1756-3305-7-150-S2.tiff]
